# Biomarkers in a Cohort of HIV-Infected Patients Single- or Co-Infected with HTLV-1, HTLV-2, and/or HCV: A Cross-Sectional, Observational Study

**DOI:** 10.3390/v14091955

**Published:** 2022-09-03

**Authors:** Adele Caterino-de-Araujo, Karoline R. Campos, Luanda M. S. Oliveira, Paula O. Rigato

**Affiliations:** 1 Laboratório de Pesquisa em HTLV, Centro de Imunologia, Instituto Adolfo Lutz, Av. Dr. Arnaldo 351, 11 Andar, Pacaembu, São Paulo 01246-000, Brazil; 2 Laboratório Estratégico, Centro de Respostas Rápidas, Instituto Adolfo Lutz, Av. Dr. Arnaldo 351, 10 Andar, Pacaembu, São Paulo 01246-000, Brazil; 3 Laboratório de Investigação Médica (LIM-56), Hospital das Clínicas, Faculdade de Medicina, Universidade de São Paulo, Av. Dr. Enéas Carvalho de Aguiar 470, Cerqueira César, São Paulo 05403-000, Brazil; 4 Laboratório de Imunologia Celular, Centro de Imunologia, Instituto Adolfo Lutz, Av. Dr. Arnaldo 351, 11 Andar, Pacaembu, São Paulo 01246-000, Brazil

**Keywords:** HIV, HTLV-1, HTLV-2, HCV, outcomes in co-infections, biomarkers, cytokines, chemokines, CD4 and CD8 cell count, HIV viral load

## Abstract

HIV, HTLV-1/-2, and HCV share routes of transmission, and such virus co-infections could account for worse outcomes of associated diseases. Measuring cytokines/chemokines, CD4 and CD8 T cells, and HIV viral load (VL) in HIV single-infected and co-infected individuals has prognostic value. We analyzed such biomarkers in 129 blood samples of HIV-infected individuals matched for age and sex and divided into six groups (G1 (69 HIV); G2 (9 HIV/HTLV-1); G3 (6 HIV/HTLV-2); G4 (11 HIV/HCV); G5 (19 HIV/HCV/HTLV-1); and G6 (15 HIV/HCV/HTLV-2)). Eight cytokines/chemokines from fifteen analytes could be compared. The highest levels of Th1 and pro-inflammatory cytokines were detected in G2 (IFN-γ) and G6 (IL-6 and IL1-β) and of chemokines in G1 (MIG, IP10, RANTES), G4 (MCP1), and G6 (MIP1-β). The highest CD4 cells number and the lowest HIV VL were identified in G3 and the opposite results in G2. Positive correlations between CD4 and CD8 cells counts and IL-6 levels were detected in G2 and G5 and of HIV VL and RANTES in G4. Negative correlations were detected between CD8 and IFN-γ in G4 and HIV VL and RANTES in G6. Despite the small number of the cohort analyzed, and although the cross-sectional study design does not allow firm conclusions, the homogeneity of the characteristics of HIV/HTLV-co-infected individuals regarding age, time and route of HIV acquisition, and criteria for introducing ART enable us to suggest a negative impact of HTLV-1 and a possible protective role of HTLV-2 in HIV infection progression in such patients.

## 1. Introduction

In the early 1980s, the first three human retroviruses were identified: human T-lymphotropic virus 1 and 2 (HTLV-1 and HTLV-2) and human immunodeficiency virus (HIV) [1,2], along with the hepatitis C virus (HCV) [3].

Since the human retroviruses share routes of transmission, HIV/HTLV co-infections occur and were more frequently detected in vulnerable populations such as intravenous drug users (IDU) [4]. IDU also have an increased risk of acquiring HCV infection, as the main route of HCV acquisition is the parenteral, and specifically in such population during drug addiction [4,5,6].

There are several possibilities of association between these four viruses, and each of them has different diseases outcomes. For instance, HIV-1 and HTLV-1 co-infection and HIV-1 and HTLV-1/-2 triple infection were associated with shorter survival, higher mortality rate, and faster progression to death, while co-infection with HIV-1 and HTLV-2 seems to have association with longer survival, slower AIDS progression, and lower mortality rate [7,8,9].

In general, HCV/HTLV-1 co-infection has been associated with the worse outcome of HCV infection worldwide (higher HCV viremia, lower rate of sustained virological response to α-interferon treatment, increased risk of chronic liver disease and cancer) except in Brazil [10]. In Brazil, HCV/HTLV-1 co-infection has been associated with the spontaneous clearance of HCV and with low liver injury [11,12,13,14] although decreases in HCV clearance were described in studies conducted in Japan and São Paulo, Brazil [15,16,17].

Curiously, one study conducted in the USA in HIV/HTLV-2-co-infected IDU showed increase in the chronic form of hepatitis C, development of liver diseases, and death due to liver cancer [11], while in São Paulo, Brazil, minor HCV viral load (VL) and spontaneous clearance of HCV were described in HIV/HTLV-2-co-infected individuals [16,17]. These contradictory results found by different groups make more studies necessary, especially regarding the interactions of these viruses and their impact on the immune response [9,18].

Oo et al. showed that HTLV-1 and HTLV-2 infections induced an innate immune response against HIV in HIV/HTLV co-infections, increasing the production of β-chemokines in peripheral blood mononuclear cells (PBMC), which could interfere with CCR5/HIV binding, thus delaying the progression to AIDS [19]. Higher levels of systemic α-chemokines (MIG and IP10) have been detected in HTLV-1-infected individuals with HAM/TSP or dermatological diseases compared to asymptomatic carriers [20,21]. Regarding cytokines, in HIV/HTLV-1-co-infected individuals, more production of Th1 cytokines (IL-2 and IFNγ) in PBMC were detected when compared to PBMC from HIV or HTLV single-infected individuals, suggesting a predominant stimulus of HTLV-1 in detriment to HIV-1 infection [22].

In São Paulo, Brazil, the four viral types circulate in patients with HIV/AIDS, providing the opportunity to study co-infections with these viruses [4,5,23]. Thus, the present study searched for systemic cytokines and chemokines in HIV single-infection and HIV/HTLV-1, HIV-HTLV-2, and HIV/HCV co-infections and in HIV/HCV/HTLV-1 and HIV/HCV/HTLV-2 triple infections in order to identify biomarkers that could better characterize such infections. In addition, using data from laboratorial parameters that monitor HIV disease progression (CD4 and CD8 T-cells count, and HIV VL levels) can ascertain which viruses’ associations have positive and negative impacts on HIV-1 infection outcome.

## 2. Materials and Methods

### 2.1. Study Design, Population, Samples, Groups for Analysis and Data Collection

This study was conducted as an anonymous, cross-sectional, descriptive, and quantitative study that employed 129 plasma samples of convenience, which were analyzed for systemic cytokines and chemokines levels. The plasma samples belonged to patients infected with HIV-1 attending the AIDS/STD Reference and Training Center in São Paulo, Brazil (CRT DST/AIDS-SP), and that participated in a study of HTLV-1 and HTLV-2 surveillance in 2015 [4]. Blood samples collected for the 2015 study were separated into plasma and PBMC, divided in aliquots, and stored at −80 °C for subsequent use. Briefly, a total of 1608 patients participated in the study: 49 had confirmed HIV/HTLV-1/2 co-infection, of which 70% were also co-infected with HCV. Of note, these co-infected patients had acquired HIV more than 25 years ago [4].

In an attempt to search for characteristic cytokine and chemokines profiles useful as biomarkers of co-infected individuals and their counterparts, we selected by convenience other 80 plasma samples from patients that resulted as HTLV-1/2-negative, matched for age and sex, and grouped these 129 samples into six groups (G) for analyses: G1 (69 HIV); G2 (9 HIV/HTLV-1); G3 (6 HIV/HTLV-2); G4 (11 HIV/HCV); G5 (19 HIV/HCV/HTLV-1); and G6 (15 HIV/HCV/HTLV-2). Demographic characteristics (age and sex) of patients as well as data of laboratorial parameters employed to monitor HIV infection (CD4 and CD8 cells count and HIV VL levels) were obtained from medical records. Of note is that the blood samples used in cytokine and chemokine measurement were collected at the same puncture site as for CD4 and CD8 cell count evaluation and HIV VL quantification. The cytokines/chemokines analyses were conducted at Instituto Adolfo Lutz (IAL) and Laboratório de Investigação Médica (LIM-56) in São Paulo, Brazil, and the CD4 and CD8 cells count and HIV VL levels at accredited laboratory of the Brazilian National HIV Laboratory Network (CRT DST/AIDS-SP). Informed consent was obtained from all subjects involved in the study, and this study was approved by the Ethics Committee for Research CTC-IAL #106D/2012 and #62H/2015 under the Brazilian Ministry of Health protocol CAAE numbers #11302512.0.0000.0059 and #52493316.1.0000.0059. The data were analyzed anonymously.

### 2.2. CD4+ and CD8+ T-Cells Immunophenotyping

Briefly, EDTA peripheral blood samples were stained using Multi-test anti-human CD45, anti-human CD3, anti-human CD4, and anti-human CD8 (BD Biosciences, San Jose, CA, USA) in Trucount tubes (BD Biosciences, San Jose, CA, USA) as recommended by the Brazilian National Network for CD4+/CD8+ T-cell Immunophenotyping. The cells types were analyzed and the absolute numbers calculated using the BD FacsCalibur flow cytometer and the Multiset software, both from Becton Dickinson™ (BD Biosciences, San Jose, CA, USA).

### 2.3. HIV-1 Viral Load Quantification

Concisely, HIV-1 RNA was quantified in a plasma sample obtained from EDTA blood collection by reverse transcription quantitative polymerase chain reaction using the Abbott RealTime HIV-1 assay (Abbott Molecular Inc., Des Plaines, IL, USA). The reaction was conducted on the automated m2000 System according to the manufacturer’s instructions (Abbott Laboratories, Des Plaines, IL, USA).

### 2.4. Cytokines and Chemokines Determination

Plasma samples used for cytokine/chemokine determinations were thawed only once and used undiluted based on previous experience of the group. The experiments were conducted in the same year of blood collection (2015). Cytometric Bead Array (CBA) kits were employed for plasmatic cytokines and chemokines quantification: Human Th1/Th2/Th17 Cytokine and Human Chemokine II (IFN-γ, TNFα, IL-2, IL-6, IL-4, IL-10 and IL-17A), and (IL-8/CXCL8, MIG/CXCL9, IP10/CXCL10, MCP-1/CCL2, and RANTES/CCL5) chemokines, joined to individuals kits Flex Set Hu for MIP-1α, MIP-1β, and IL-1β quantification, all from Becton Dickinson™ (BD Biosciences, San Jose, CA, USA). Reactions were performed following the manufacturer’s instructions; the samples were acquired in the flow cytometer Fortessa LSR (BD Biosciences, San Jose, CA, USA), and the results were analyzed by the FCAP Array Software Version 3.0 from Becton Dickinson™ (BD Biosciences, San Jose, CA, USA).

### 2.5. Statistical Analyses

After collecting the patients’ information from their medical records and from the cytometer results, a database was built using the Excel 2010 (Windows 10 software, Microsoft Corporation, Redmond, Washington, USA). Differences in the numbers of males and females in each group were evaluated statistically using the chi-square test. GraphPad Prism software version 5.03 (GraphPad, San Diego, CA, USA) was used for age, CD4 and CD8 T-cells count, and HIV VL and cytokine and chemokine levels comparisons. Kruskal–Wallis analysis of variance (ANOVA) complemented with Dunn’s multiple comparison test was employed for comparing three or more groups and Mann–Whitney U-test for two groups. Results with a *p*-value of ≤0.05 were considered statistically significant. The Spearman’s rank correlation test was employed for measure the strength of a monotonic relationship between CD4, CD8, and HIV viral load and each piece of cytokine and chemokine data. The values were denoted as ranks (r_s_) that could vary from +1 “perfect positive correlation” to −1 “perfect negative correlation”. Intermediate values from 0.00–0.19 denote “very weak”, 0.20–0.39 “weak”, 0.40–0.59 “moderate”, 0.60–0.079 “strong”, and 0.80–1.0 “very strong” correlation.

## 3. Results

**Figure 1:**
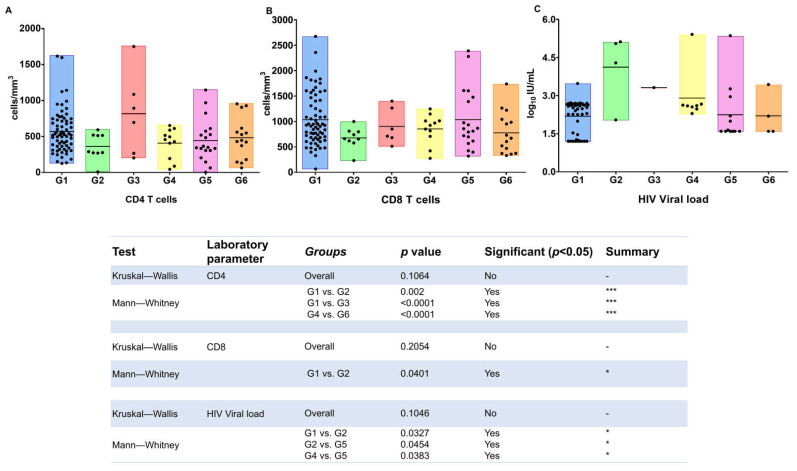
Number of CD4 and CD8 T cells and levels of HIV viral load in patients with HIV infection and HTLV-1, HTLV-2, and/or HCV co-infections. G1 (HIV), G2 (HIV/HTLV-1), G3 (HIV/HTLV-2), G4 (HIV/HCV), G5 (HIV/HCV/HTLV-1), G6 (HIV/HCV/HTLV-2). Subfigures (**A**–**C**)refer to the values of CD4 T cells, CD8 T cells and HIV viral load quantification, respectively. The horizontal line inside the bars denotes mean values. Differences statistically significant are depicted using Kruskal–Wallis analysis of variance for three or more groups and Mann–Whitney U-test for two groups. *p*-values depicted as asterisks correspond to: * *p* < 0.05; *** *p* ≤ 0.001.

The characteristics of patients and the results of the laboratorial parameters employed in monitoring HIV infection in each group are presented in Table 1. Briefly, as expected, no difference was observed according to age and sex; the mean age of groups ranged from 46 to 50 years, and both sexes were represented. Regarding CD4 T-cells count, the highest mean value was detected in HIV/HTLV-2 co-infection (G3), while the lowest mean value was found in HIV/HTLV-1 co-infection (G2). All groups had CD8 T-cells counts > 670 cells/mm^3^, with the lowest value detected in G2. In addition, G2 had the highest value of HIV VL, while the lowest value was detected in G3, emphasizing that only one sample had quantified HIV-RNA (3.31 log10 UI/mL), and in the other plasma samples of this group, the HIV VL was undetectable. Table 1 also shows the mean values and standard deviation of CD4 T cells, CD8 T cells and HIV VL, and the differences were statistically significant among the groups, while Figure 1 discloses the mean values (horizontal lines) and the scatter plot of these values for each study group.

**Figure 2:**
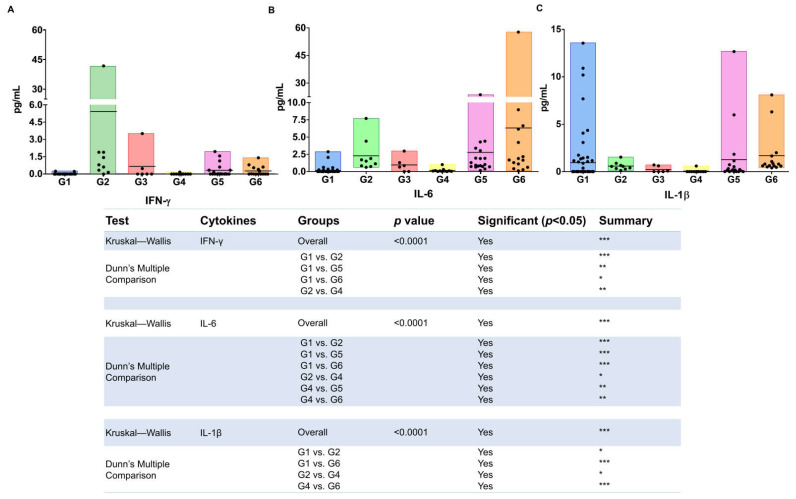
Levels of cytokines detected in plasma samples of HIV-infected and HTLV-1-, HTLV-2-, and/or HCV-co-infected patients. G1 (HIV), G2 (HIV/HTLV-1), G3 (HIV/HTLV-2), G4 (HIV/HCV), G5 (HIV/HCV/HTLV-1), G6 (HIV/HCV/HTLV-2). Subfigures (**A**–**C**) refer to the values of IF-γ, IL-6, and IL-1β quantification, respectively. The horizontal line inside the bars denotes mean values. Differences statistically significant are depicted using the Kruskal–Wallis analysis of variance (ANOVA) complemented with Dunn’s multiple comparison test. *p*-values depicted as asterisks correspond to: * *p* < 0.05; ** *p* ≤ 0.01; *** *p* ≤ 0.001.

**Figure 3:**
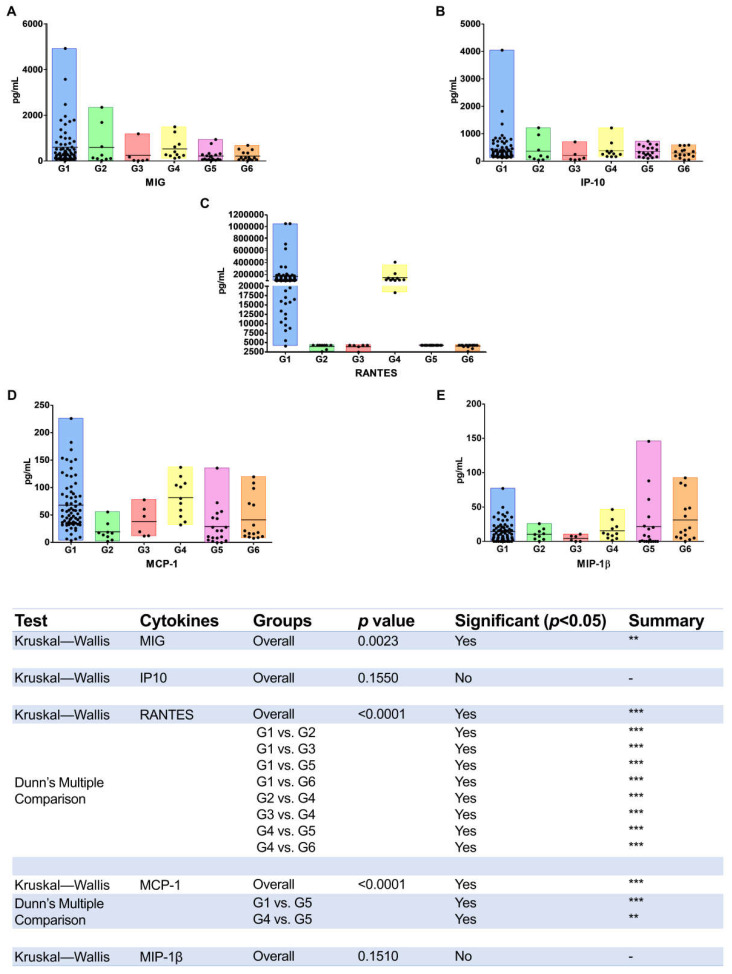
Levels of chemokines detected in plasma samples of HIV-infected and HTLV-1-, HTLV-2-, and/or HCV-co-infected patients. G1 (HIV), G2 (HIV/HTLV-1), G3 (HIV/HTLV-2), G4 (HIV/HCV), G5 (HIV/HCV/HTLV-1), G6 (HIV/HCV/HTLV-2). Subfigures (**A**–**E**) refer to the values of MIG, IP-10, RANTES, MCP-1 and MIP-1β quantification, respectively. The horizontal line inside the bars denotes mean values. Differences statistically significant are depicted using the Kruskal–Wallis analysis of variance (ANOVA) complemented with Dunn’s multiple comparison test. *p*-values depicted as asterisks correspond to: ** *p* ≤ 0.01; *** *p* ≤ 0.001.

In relation to cytokines and chemokines present in the plasma samples of such patients’ groups, in general, low levels were detected, and only eight out of fifteen analytes (cytokines and chemokines) evaluated could be compared. The results of the mean value and interquartile range (IQR—1st and 3rd) are presented in Table 2; statistically significant differences and the major mean values were highlighted. The mean values and the scatter plot of these values in each study group along with the statistical differences among groups are presented in Figure 2 and Figure 3.

Briefly, the IFN-γ, IL-6, and IL-1β were the three cytokines that could be analyzed, with the major levels detected in G2 (IFN-γ) and G6 (IL-6 and IL-1β) groups (Table 2). Differences statistically significant in IFN-γ levels among groups are depicted in Figure 2A, emphasizing minor level of such cytokine in G1 (HIV single infection) and the major in G2 (HIV/HTLV-2). The highest levels of IL-6 and IL-1β were found in HIV/HCV/HTLV-2 (G6, Table 2). Regarding IL-6, the minor values were detected in G1 and G4 (HIV and HIV/HCV), with differences statistically significant compared to the other groups (Figure 2B), and concerning IL-1β, the minor value was detected in G4 (HIV/HCV) and the major values in G6 and G1, with differences statistically significant (Figure 2C).

In relation to α-chemokines, MIG and IP10 had the highest levels detected in G1 (HIV single infection) (Table 2), but only MIG disclosed differences statistically significant among groups (Figure 3A,B). The MCP-1 chemokine was detected in higher concentrations in HIV and HIV/HCV (G1 and G4), with statistically significant differences only when they were compared to G5 (HIV/HCV/HTLV-1) (Figure 3D).

Regarding the β-chemokines, the RANTES was the chemokine that presented the highest values in relation to the others chemokines, mostly in G1 and G4 (HIV and HIV/HCV, respectively), with differences statistically significant when compared with the others groups (Figure 3C). The highest mean value of MIP-1β was detected in G6 (HIV/HCV/HTLV-2 co-infection) and the lowest mean value in G3 (HIV/HTLV-2 co-infection) (Table 2) but, overall, without statistical differences among groups (Figure 3E).

Regarding the search for any correlation between the values of CD4, CD8, and HIV VL and the cytokines and chemokines concentrations using the Spearman’s correlation test, a strong positive monotonic correlation between CD4 and CD8 cells counts and IL-6 levels was detected in G2 (CD4, r_s_ = 0.717, *p* = 0.037; and CD8, r_s_ = 0.655, *p* = 0.056) and a moderate positive monotonic correlation of CD8 cell numbers and IL-6 in G5 (r_s_ = 0.536, *p* = 0.018). In G4, a negative correlation between CD8 cells count and IFN-γ was detected (r_s_ = −0.656, *p* = 0.028). Concerning correlation between HIV VL levels and cytokines and chemokines concentrations, RANTES was the unique chemokine that presented correlation: a very strong positive monotonic correlation was detected in G4 (r_s_ = 0.915, *p* = 0.001) and a strong negative correlation in G6 (r_s_ = −0.990, *p* = 0.010).

## 4. Discussion and Conclusions

HIV/HCV, HIV/HTLV-1, HCV/HTLV-1, and HIV/HCV/HTLV-1 co-infections have been associated with poor prognosis, such as rapid progression to liver disease, neuropathies or AIDS, and/or increased risk of morbidity and mortality when compared with the mono-infections counterparts [7,9,10,15,18,24]. Little is known concerning the interaction of HTLV-2 in HCV infection, and the results available in the literature are controversial, and those that address triple infections are even rarer [6,15,16,17,18].

Since the balance of the immune response to such viruses can predict the clinical status of patients (asymptomatic or symptomatic), especially in HTLV-1 and HCV infections in which tissue damage (HAM/TSP or liver disease) seems to be related to the exacerbated stimulus either by inflammatory or cytotoxic processes [15,25,26], it is useful measure cytokines and chemokines in plasma samples from patients with HIV single infection and with HCV, HTLV-1, or HTLV-2 co-infections in an attempt to identify biomarkers characteristic of each viruses association and/or to highlight higher inflammatory processes that could be causing damage to the host tissues.

In the present study, it was not possible to assess the impact of co-infections on the progression of the hepatitis C (HCV VL, HCV clearance, and other liver functional laboratorial analysis); however, at the time of blood collection, there are no reports of diseases progression in these patients’ medical records. It is noteworthy that almost all patients with HIV/HTLV-1/-2 co-infections (47/49) regardless of whether or not they had HCV infection were on antiretroviral therapy (ART) [4], while in HIV single infection (G1) and HIV/HCV co-infection (G4), 55% and 36.4%, respectively, were on ART. Nevertheless, there was no difference in the results analyzed in the present study when G1 and G4 were separated into ART and naïve groups (data not shown), so the groups were kept back for statistical analysis.

Interestingly, the highest HIV VL was detected in HIV/HTLV-1 co-infection, which is in agreement with data from the literature that disclose worse outcomes and death from AIDS in HIV/HTLV-1-co-infected patients [7,8,9]. All the other groups had a mean HIV VL < 3.0 log10 UI/mL, highlighting that only one patient in G3 (HIV/HTLV-2 co-infection) had detectable HIV-RNA, which corroborates data from the literature of slow progression to AIDS in HIV/HTLV-2-co-infected individuals [8,27]. These results emphasize and agree with the negative impact of HTLV-1 and the protective role of HTLV-2 in HIV/AIDS disease progression [8,27] and show that HCV in HIV-infected individuals does not influence HIV replication. On the other hand, there are reports showing that HIV infection interferes with HCV replication, increasing in 0.51 log10 the HCV VL in HIV/HCV co-infection [16,17], but this could not be evaluated in the present study, as we did not have access to HCV VL data from these patients.

Different from expected regarding the increased number of CD4 T cells in HIV/HTLV-1-co-infected individuals due to lymphoproliferation [7,8], in the present study, a minor number of such cells was detected in this group of patients (G2). This finding can be related to the long time of HIV infection (more than 25 years) and probably the delay in starting anti-retroviral therapy (ART) in such patients, making it impossible to recover the CD4 T cells lost. In fact, CD4 cells count was widely used in the past as a surrogate marker to define the moment to start ART. Further, the artificial increment in CD4 cells count can cause a delay in introduction of ART, increasing the risk of AIDS-related events and death [28]. Therefore, recommendation for considering earlier initiation of ART in persons with HIV/HTLV-1 co-infection has been described [29] and proven to be successful in normalizing survival time in these co-infected patients [28]. Taking together the above information, we could hypothesize a delay in starting ART in such patients’ group. Supporting the negative impact of HTLV-1 in HIV disease progression, herein, a strong correlation of CD4 and CD8 cells counts and IL-6 levels were detected in such group of patients. Of note, no case of HTLV-1-associated disease was detected in HIV/HTLV-co-infected patients of this study.

Moreover, corroborating the protective role of HTLV-2 in HIV infection, the highest CD4 T-cells counts were obtained in G3, which, although ART was started using the same criteria for HIV/HTLV-1-co-infected individuals, was able to better control the HIV VL, consequently keeping stable the number of CD4 cells. The mechanisms by which the HTLV-2 controls HIV replication and AIDS disease progression were largely discussed by Casoli et al. [27]. Briefly, one of them is the HTLV-2 transcriptional activating gene known as Tax2, which is, among others, responsible for induction of CC-chemokines (MIP-1a/CCL3, MIP-1b/CCL4, and RANTES/CCL5) that play a major role in innate immune responses against HIV-1. These chemokines (especially CCL3L1 isoform) bind the CCR5 HIV-1 co-receptor promoting its internalization, thus preventing cells infection [27]. Unfortunately, no correlation between HIV VL and any cytokines and chemokines could be proven in HIV/HTLV-2-co-infected individuals in the present study (all except one had HIV-RNA detectable), but a negative correlation between HIV VL and RANTES was detected in HIV/HCV/HTLV-2-co-infected individuals.

Regarding the quantification of cytokines and chemokines in the plasma samples of the present study, it is worth mentioning the difficulty in comparing the results obtained with those described in the literature, mostly because of the different stages and time of HIV infection of patients and systems/reagents employed in analyses. Although plasma samples are the best biological specimens to be analyzed, as they represent what is being released at that moment into circulation and were employed in several studies [18,20,21,25,30], stimulated cell culture supernatants are also used in other studies [19,22], however the results in different biological samples and with different systems/reagents such as ELISA kits [19,21,22,30] or LUMINEX [18] instead of CBA [20,25] do not allow comparative results analyses with the current study.

Three cytokines showed higher concentrations in HTLV-1/-2-co-infected groups. IL-6 was mainly detected in HIV/HCV/HTLV-2 triple infection, and despite having anti- and pro-inflammatory activity and activating/maturating neutrophils, macrophages, and natural killer (NK) cells [22], its role in co-infections is unclear. There are reports of constant concentrations of IL-6 and of the IL-1β induction in plasma of untreated individuals with chronic HIV infection [31], and curiously, these cytokines were also detected mainly in samples of HIV/HCV/HTLV triple infections in the present study. These results are in agreement with those found also by Brites et al. that associated higher levels of IL-1β in patients with HIV/HCV/HTLV-1 co-infection and with HCV RNA clearance [18]. Herein, we detected a strong positive correlation between CD4 and CD8 cells counts and IL-6 levels in HIV/HTLV-1 and HIV/HCV/HTLV-1 co-infections, emphasizing more inflammation in HTLV-1-co-infected individuals. Although IL-6 had an essential role in the acute phase of immune reactions, constant high levels of this cytokine cause pathological effects in chronic infections [32]. Recently, it was shown that IL-6 is a strong predictor of adverse outcomes in HIV infection—more than C-reactive protein or d-dimers [33].

Another cytokine with potent Th1 activity and that showed higher levels in HIV/HTLV-1 co-infection was IFN-γ, which is in agreement with the findings of other studies conducted in culture supernatant [22], and plasma [18]; they pointed to HTLV-1 as the main inducer of the Th1 response in HIV/HTLV-1 dual infection. In addition, Abrahão et al. [22] associated high concentrations of IFN-γ with asymptomatic carrier status in HTLV-1 infection. In other studies, IFN-γ secretion was associated with the production and increase in plasma levels of CXCL9/MIG and CXCL10/IP10 in individuals that developed HAM/TSP [21,25]. However, in the present study, no association could be done since no case of HAM/TSP was detected, and the highest concentrations of the two chemokines (MIG and IP10) were found in HIV single infection, which is precisely the group with the lowest concentration of IFN-γ (0.01 pg/mL). This result could mean a negative feedback in an attempt to balance the immune response in HIV group, as previously described [21]. On the other hand, a negative correlation of HIV VL and IFN-γ was detected herein in HIV/HCV-co-infected patients.

In HIV-1-infected individuals, the IP-10 levels were mainly elevated [34,35] and are associated to higher progression of HIV-1 infection [36]. In the present study, lower IP-10 levels were detected in HIV/HTLV-2-co-infected patients, suggesting that HTLV-2 promoted less inflammation and lower levels of this and other pro-inflammatory cytokines and chemokines systemically.

In relation to CCL2/MCP1 that has strong chemotactic activity of monocytes, NK cells, and CD4 T cells [34], it was detected in the present study at a higher concentration in HIV/HCV co-infection, with statistical difference only with HIV single infection. A high level of this CC-chemokine was associated with favoring HIV replication as a result of the recruitment of CCR2+ cells to new “rounds” of the replicative cycle of this virus [37]. However, the HIV VL data of the studied samples do not allow this conclusion since the mean HIV VL of all groups was below 3.0 log10 UI/mL except for G2.

CCR5 ligands have long been studied in HIV infection, with CCL5/RANTES as the main natural suppressor of HIV infection, secreted by activated CD8 T lymphocytes. In acute HCV infection, high plasma levels of RANTES were associated with the recruitment of effector CD4 and CD8 T cells, contributing to viral clearance [25]. It is noteworthy that in the present study, the highest levels of RANTES were detected in HIV-single-infection and HIV/HCV co-infection. Oo et al. measured chemokines in patients with HIV single infection and in patients with HIV/HTLV-1 and HIV/HTLV-2 co-infections and detected an increase in CCL3/MIP-1α, CCL4/MIP-1β, and CCL5/RANTES, especially in HTLV-2-co-infected individuals. This pattern was maintained when lymphocytes in culture were stimulated with the HTLV-2 Tax2, supporting that HTLV-2 stimulates the production of chemokines that can modify the interaction of HIV with its co-receptors, mainly CCR5 [19].

Although the stimulation of CC-chemokines by HTLV-1/-2 was highlighted, the highest concentration of RANTES was observed in the present study in HIV single infection and the highest level of MIP-1β in the HIV/HCV/HTLV-2 triple infection. Interestingly, the lowest concentrations for both MIP-1β and RANTES were found in HIV/HTLV-2 co-infection. Taking into account the results previously exposed [19], we could suppose that the low concentrations of MIP-1β and RANTES in the HIV/HTLV-2 group may be related to the binding of these proteins to their respective receptors (CCR1, CCR3, and CCR5 preferentially), preventing its detection in plasma, and consequently blocking the entry of HIV R5 strains and decreasing replication of HIV RNA. Curiously, and in agreement with this hypothesis, a negative correlation between HIV VL and RANTES was detected herein in HIV/HCV/HTLV-2-co-infected patients. Of note, another study of the HIV tropism in HIV/HTLV co-infection that included patients of the present study showed that in HIV/HTLV-1 co-infection, there were more HIV X4 strains and in HIV/HTLV-2 co-infection more HIV R5 strains, with statistically significant difference, *p* = 0.038 (data not shown, sequences deposited in GenBank, Accession Numbers KT343383 to KT343424). Altogether, the results obtained in HIV/HTLV-2-co-infected individuals of the present study agree with the reports from Casoli et al. [27], Turci et al. [38], Pilotti et al. [39], and Ruiz-Mateos et al. [40], who pointed a protective role of HTLV-2 in HIV infection outcome.

Worth mentioning is that the CBA technology employed in the present study allows multiple cytokines and chemokines quantifications in individual samples, showing higher reproducibility and sensitivity than ELISA [41]. Multiplexed technologies reduce cost, time, and labor compared to single-reaction-based detection methods such as ELISA, and it has been the chosen assay for biomarker or cytokine signature discovery in several biomedical sciences [42]. In the present study, although seven of the fifteen cytokines and chemokines were found to be undetectable by the CBA kits, this could be due to the diverse therapies that the patients were undergoing rather than the high dilution or degradation of the sample since the samples were thawed only once and tested undiluted.

Despite the fact that the present work has a series of limitations in relation to the small samples size in each group (which is reflected in the low incidence of HIV, HTLV-1 or HTLV-2, and HCV double or triple infection), the cross-sectional design (only a single quantification analyses), the absence of data on the right moment of infection by each agent, and the possible bias in the interpretation of results, this study is important since it was conducted in a period when the introduction of ART took into account the number of CD4+ cells, making this cohort of patients of utmost interest. At present, ART is initiated soon after HIV-1 diagnosis as well as HIV pre-exposure prophylaxis, making this kind of population never more available for analyses. In addition, this work discloses the complexity of the immune response when it involves more than one virus and highlights the need of further studies, especially in triple infections and co-infections by HTLV-2, to confirm or refute the results obtained and the raised hypotheses. In this sense, it is up to the countries and regions where such viruses circulate to carry out these studies, as is the case of São Paulo, Brazil.

In summary, the present study shows that HIV/HTLV-1- and HIV/HTLV-2-co-infected patients that had acquired HIV infection 25 years ago and that were on ART disclose more plasmatic Th1 and pro-inflammatory cytokines, while patients with HIV single infection and co-infected with HCV disclose more plasmatic chemokines. IP10, RANTES, and MIG were the chemokines mostly detected in HIV single infection, IFN-γ in HIV/HTLV-1 co-infection, MCP-1 in HIV/HCV co-infection, IL-6, IL-1β, and MIP-1β in HIV/HCV/HTLV-2 co-infection and, interestingly, minor levels of MIP-1β in HIV/HTLV-2 co-infection. Strong positive correlation between CD4 and CD8 cells counts and IL-6 levels was detected in HIV/HTLV-1 co-infection, between CD8 cells count and IL-6 levels in HIV/HCV/HTLV-1 co-infection, and very strong correlation between HIV VL and RANTES in HIV/HCV co-infection. On the other hand, strong negative correlations between CD8 cells count and IFN-γ levels were detected in HIV/HCV co-infection and very strong negative correlation between HIV VL and RANTES in HIV/HCV/HTLV-2 co-infection. Of note, this is the first time that fifteen cytokines and chemokines were measured in HIV single-infected and HTLV-1-, HTLV-2-, and HCV-co-infected patients in São Paulo, Brazil, and the first study that shows the possible benefit of HTLV-2 in protecting patients from HIV infection progression in this country.

## Figures and Tables

**Table 1:** Demographic characteristics, CD4 and CD8 T-cells counts, and HIV viral load in patients with HIV infection and HTLV-1, HTLV-2, and/or HCV co-infections.

Groups	Age * (Years)	Sex				
		Male	Female	CD4 **	CD8 **	HIV Viral Load **
		*n* (%)	*n* (%)	Cells/mm³	Cells/mm³	log_10_ IU/mL
G1 (*n* = 69)	46.0 (31–65)	39 (57.4)	29 (42.6)	571.6 (±296.5) ^a,b^	1034.0 (±510.6) ^d^	2.19 (±0.64) ^e^
G2 (*n* = 9)	48.2 (28–63)	4 (44.5)	5 (55.5)	363.7 (±187.0)	675.6 (±209.9)	4.12 (±1.43) ^f^
G3 (*n* = 6)	46.2 (41–50)	1 (16.7)	5 (83.3)	818.3 (±575.2)	902.5 (±350.9)	3.31 (0.00) ***
G4 (*n* = 11)	48.2 (36–59)	5 (45.4)	6 (54.6)	409.6 (±209.7) ^c^	851.9 (±291.7)	2.90 (±1.02) ^g^
G5 (*n* = 19)	50.3 (42–66)	12 (63.2)	7 (36.8)	444.7 (±293.7)	1037.0 (±597.4)	2.24 (±1.13)
G6 (*n* = 15)	50.7 (43–61)	8 (53.3)	7 (46.7)	484.3 (±287.7)	774.8 (±414.5)	2.20 (±0.87)

Legend: G1 (HIV), G2 (HIV/HTLV-1), G3 (HIV/HTLV-2), G4 (HIV/HCV), G5 (HIV/HCV/HTLV-1), G6 (HIV/HCV/HTLV-2); * mean (minimum and maximum), ** mean (±standard deviation), *** data from only one sample, the others resulted below the detection limit of the test. Significant *p*-values were found in: ^a^ *p* = 0.002 G1 vs. G2; ^b^ *p* < 0.0001 G1 vs. G3; ^c^ *p* < 0.0001 G4 vs. G6; ^d^ *p* = 0.0401 G1 vs. G2; ^e^ *p* = 0.0327 G1 vs. G2; ^f^ *p* = 0.0454 G2 vs. G5; ^g^ *p* = 0.0383 G4 vs. G5.

**Table 2:** Cytokines and chemokines detected in plasma samples of HIV-infected and HTLV-1-, HTLV-2-, and/or HCV-co-infected patients.

Cytokines and Chemokines * pg/mL	Groups						
	G1 (*n* = 69)	G2 (*n* = 9)	G3 (*n* = 6)	G4 (*n* = 11)	G5 (*n* = 19)	G6 (*n* = 15)	*p* **
**IFN-γ**	0.01	**5.42**	0.66	0.02	0.33	0.27	**<0.0001**
	(0.0–0.0)	(0.25–1.89)	(0.0–1.24)	(0.0–0.0)	(0.0–0.34)	(0.0–0.55)	
**IL-6**	0.16	2.28	0.96	0.17	2.78	**6.32**	**<0.0001**
	(0.02–0.14)	(0.78–3.15)	(0.0–1.72)	(0.01–0.14)	(0.73–3.04)	(0.62–6.15)	
**IL-1β**	0.95	0.59	0.23	0.05	1.27	**1.69**	**<0.0001**
	(0.0–0.19)	(0.30–0.77)	(0.0–0.64)	(0.0–0.0)	(0.0–0.73)	(0.59–1.65)	
**MIG**	**601.2**	593.9	241.8	526.8	215.3	213.8	**0.0023**
	(125.9–722.4)	(89.23–1154)	(14.10–425.3)	(223.9–729.2)	(56.71–274.9)	(49.66–354.0)	
**IP-10**	**450.0**	366.6	211.2	382.3	360.3	302.9	0.155
	(209.4–506.2)	(71.92–685.4)	(63.47–365.9)	(175.2–366.3)	(164.3–545.2)	(182.5–407.0)	
**MCP-1**	68.12	19.55	38.45	**81.82**	29.24	41.44	**<0.0001**
	(35.24–88.43)	(7.77–26.61)	(12.32–65.04)	(47.75–107.9)	(4.66–45.30)	(11.06–71.21)	
**MIP-1β**	14.32	10.45	4.51	15.61	21.66	**31.32**	0.151
	(3.53–21.35)	(3.33–16.41)	(0.0–8.47)	(4.95–21.57)	(0.0–22.38)	(4.98–48.76)	
**RANTES**	**102549**	4049	3996	82512	4366	4148	**<0.0001**
	(20768–90364)	(3786–4366)	(3636–4366)	(34444–72660)	(4366–4366)	(4033–4366)	

Legend: G1 (HIV), G2 (HIV/HTLV-1), G3 (HIV/HTLV-2), G4 (HIV/HCV), G5 (HIV/HCV/HTLV-1), G6 (HIV/HCV/HTLV-2). * mean (interquartile range (IQR, 1st and 3rd)), ** ANOVA—Kruskal–Wallis test.

## Data Availability

The data analyzed in this study are included within the paper.
